# Editorial: The role of microbial glycosylation in host-pathogen interactions

**DOI:** 10.3389/fcimb.2025.1689780

**Published:** 2025-09-09

**Authors:** Kelly M. Fulton, Susan M. Twine, Christopher W. Reid

**Affiliations:** ^1^ Human Health Therapeutics Research Centre, National Research Council Canada, Ottawa, ON, Canada; ^2^ Department of Biology, Faculty of Science, Carleton University, Ottawa, ON, Canada; ^3^ Department of Biological and Biomedical Sciences, Bryant University, Smithfield, RI, United States

**Keywords:** glycosylation, host-pathogen interactions, microbial virulence factors, infectious diseases, carbohydrates

There are over a trillion microbial species on Earth, including bacteria, viruses, fungi, and parasites. These organisms are typically too small to be seen with the naked eye but still have significant impacts on the world around them. While the vast majority of microorganisms are harmless or even beneficial to humans, a small proportion are considered pathogenic. Though they represent less than 1% of all microbes (Microbiology by numbers, 2011), these pathogens pose a real and ongoing threat to human welfare with sepsis alone being responsible for over 20% of deaths globally each year (Rudd et al., 2020; Ikuta et al., 2022). Infectious diseases also indirectly compromise the economy by putting strain on health care systems and reducing labour productivity.

What distinguishes a disease-causing microbe from a benign microbe is predominantly linked to the nature of specific host-pathogen interactions. The net result of offensive and defensive mechanisms on both sides of this dynamic relationship determines the severity of pathogenesis. The collection of attributes enabling pathogenesis of microorganisms are called virulence factors. These include the capacity for motility, adherence to and invasion of host cells, host immune system stimulation or evasion, physical defense, toxin production, and so on. Many of these virulence factors depend upon glycoconjugate macromolecules (Yakovlieva et al., 2021), as do many of the defensive host mechanisms, making the host-pathogen glycome a key determinant of disease progression (Lin et al., 2020). Not surprisingly, most host-pathogen interactions occur at the cell surface interface. Microbial surfaces are often decorated with a variety of carbohydrate structures that play critical roles in these interactions. This special Research Topic presents both a review of existing knowledge and original research concerning the role of carbohydrates in host-pathogen interactions and pathogenesis, providing insight that may lead to novel therapeutic options.

*Toxoplasma gondii* is an intracellular parasite that usually causes asymptomatic infection in immunocompetent individuals but can lead to toxoplasmosis in those who are immunocompromised. It exists as either tachyzoites (during acute infection) or bradyzoites within cysts (during latent chronic infection) and can convert between the two stages of the life cycle. The cyst wall, which is made up of polysaccharides such as chitin, is a physical barrier that protects the bradyzoites against host responses and its regulation is integral to the parasitic life cycle. Bando et al. generated a knockout mutant of *T. gondii* that lacked the hypothetical chitinase-like protein 1 (a chitin degrading enzyme). This mutant had reduced capacity for reactivation from bradyzoite to tachyzoite, demonstrating the critical and dynamic role of surface carbohydrates in host-pathogen interactions and subsequent disease progression. An improved understanding of the mechanisms regulating acute and chronic infection may provide opportunities for therapeutic development.

Many bacterial pathogens possess an outer capsular polysaccharide (CPS) layer that serves to protect the cell from the external environment, which may include host defenses and bacteriophages. Some bacteriophages express capsule depolymerase enzymes that degrade CPS to facilitate receptor binding. Wang et al. recently identified and expressed a putative depolymerase enzyme (Depo27) from *Acinetobacter pittii*, a nosocomial and antimicrobial resistant (AMR) pathogen. Following screening of several clinical isolates, they demonstrated that Depo27 degraded the capsules of *A. pittii* 7 and 1492. These isolates were subsequently susceptible to human serum-mediated killing. Leveraging this natural mechanism for overcoming the capsule defense of AMR bacteria holds promise for the development of novel biologic-based therapies.

Gram-negative bacteria elaborate a lipopolysaccharide (LPS) layer. In *Helicobacter pylori*, the biosynthetic pathways responsible for LPS and glycosylation of surface proteins have significant overlap. Barrett et al. investigated the impact of several mutations within these pathways on host immune stimulation. They observed that glycosylation mutant strains producing truncated LPS structures resulted in a reduced innate immune response (reduced secretion of interleukin 8 compared to wildtype). Interestingly, the truncated LPS did not alter the immature dendritic cell maturation, suggesting that the truncation did not reduce the adaptive immune response. Based on these findings, there may be opportunities to target glycan biosynthesis for therapeutically advantageous outcomes by simultaneously inhibiting the innate response (that causes chronic inflammation leading to gastritis, ulcers, and cancer) and maintaining the adaptive immune response.

*Porphyromonas gingivalis*, a Gram-negative anaerobe, is an important species involved in periodontitis disease progression. However, the pathoadaptive mechanisms that allow this organism to thrive in the host environment are poorly understood. In the work presented by Ghods et al., the cellular second messenger cyclic-di-adenosine monophosphate (c-di-AMP) regulates the structure of LPS in this organism. To investigate this relationship, they used a combination of fast lipid analysis technique (FLAT) with MALDI trapped ion mobility spectroscopy time of flight mass spectrometry (MS), which allowed for direct biomass MS or MS/MS visualization and analysis of Lipid A variants. The reported findings demonstrated that c-di-AMP serves as a metabolic hub linking the bioenergetic state of the cell to changes in LPS composition, including glycosylation and fatty acid patterns. Changes in c-di-AMP levels resulted in significant shifts in LPS structure and immunostimulatory potential. Given that c-di-AMP is unique to bacteria and crucial to *P. gingivalis* survival, targeting components of the c-di-AMP network could serve as a viable anti-infective strategy for periodontal disease.

Many bacterial surface and transmembrane proteins in both Gram-positive and Gram-negative species, such as S-layers, flagellins, and pilins, are glycoproteins. The glycosylation may be through either *N-* or *O-*linkage and often incorporates unique carbohydrates not found in eukaryotic organisms. Though the glycans are known to serve diverse functions with respect to host-pathogen interactions, it must also be noted that these glycans can alter the immunogenicity of the proteins they modify. Focusing on *O-*linked glycosylation of pilin in *Neisseria*, Børud and Koomey have reviewed the impact of glycan heterogeneity and phase variable expression of glycosylation related genes in host immune evasion and survival. This antigenic variability may be responsible for a lack of natural protection following *Neisseria* infection, and will be an important consideration for vaccine development.

Microbial surface glycoconjugates are structurally and functionally diverse. This Research Topic showcases that diversity through the breadth of studies included and further highlights the critical involvement of carbohydrates in many microbial virulence mechanisms. Together with a growing body of literature, these studies continue to unravel the extent to which microbial glycans engage with the host immune system, influencing the severity of disease. Understanding the role of variable and dynamic glycan-mediated host-pathogen interactions is critical for the development of future therapeutics and vaccines to reduce the burden of infectious diseases.
